# Seabirds and native vegetation drive ecosystem processes that support atoll island resilience

**DOI:** 10.1126/sciadv.aei1145

**Published:** 2026-10-14

**Authors:** Nicholas A. J. Graham, Cassandra E. Benkwitt, Ines D. Lange, Chris T. Perry, Eleanor R. Thomson, Lisa M. Wedding, Ruth E. Dunn, Sebastian Steibl, Courtney E. Stuart, Laura-Li Jeannot, Jennifer Appoo, Julia Rodriguez Fillol, Marleen Stuhr, Pirta Palola, Peter Carr, Jayna L. DeVore, Simon Ducatez, Anna Zora, Ayla K. Hastings, Solène Fabre Barroso, Sean Evans, Melissa Schulze, Emma Cotton, Hazel Reddington, Yadvinder Malhi

**Affiliations:** ^1^ Lancaster Environment Centre, Lancaster University, Lancaster, UK.; ^2^ California Oceans Program, The Nature Conservancy, CA, USA.; ^3^ Geography, Faculty of Environment, Science and Economy, University of Exeter, Exeter, UK.; ^4^ School of Geography and the Environment, University of Oxford, Oxford, UK.; ^5^ The Lyell Centre, Heriot-Watt University, Edinburgh, UK.; ^6^ Centre d'Ecologie Fonctionnelle et Evolutive, 1919 Route de Mende, 34293 Montpellier, France.; ^7^ School of Biological Sciences, University of Auckland, Auckland, New Zealand.; ^8^ Naturalis Biodiversity Center, Leiden, Netherlands.; ^9^ UMR ENTROPIE, Université de La Réunion, La Réunion, France.; ^10^ Blue Economy Research Institute, University of Seychelles, Anse Royale, Mahé, Seychelles.; ^11^ Leibniz Centre for Tropical Marine Research (ZMT), Fahrenheitstraße 6, Bremen, Germany.; ^12^ Chagos Conservation Trust, London, UK.; ^13^ Tetiaroa Society, Teti’aroa, French Polynesia.; ^14^ University of French Polynesia, IFREMER, ILM, IRD, UMR SECOPOL, Campus d'Outumaoro, Punaauia, Tahiti, French Polynesia.; ^15^ IRD, ILM, IFREMER, UPF, UMR SECOPOL, Campus d'Outumaoro, Punaauia, Tahiti, French Polynesia.; ^16^ Frégate Island Foundation, Roche Caiman, Mahé, Seychelles.; ^17^ Fenua Ecologie, 98702 Faaa Centre, Tahiti, French Polynesia.; ^18^ Cousine Island Conservation, Cousine Island, Seychelles.; ^19^ Island Conservation Society, Pointe Larue, Mahé, Seychelles.; ^20^ Leverhulme Centre for Nature Recovery, University of Oxford, Oxford, UK.

## Abstract

Ecosystem restoration is expected to enhance ecosystem resilience to climate change, but few empirical and mechanistic demonstrations of such assertions exist. Here, we explore this topic for tropical atoll systems, combining science on nutrient subsidies and geo-ecological functions through a unifying ecological energetics lens. We show that native forest cover and seabird nutrient inputs increase primary productivity and secondary metabolism on islands and adjacent coral reefs, scaling with key ecosystem processes. We then demonstrate that seabird and native forest restoration could contribute to reducing island climate change vulnerability by (i) increasing reef accretion rates such that they keep pace with sea-level rise under a 2.0°C warming scenario, (ii) enhancing the production rate of sediment, which could contribute to island maintenance, and (iii) increasing vegetation fine root length, aiding island sediment stabilization. Collectively, our findings provide an integrated energetics and geo-ecological perspective that suggests rewilding of atolls can contribute to climate resilience.

## INTRODUCTION

Atoll islands constitute the most numerous island type in the Indo-Pacific. They provide the only landmass for multiple countries, are surrounded by ecologically and societally critical coral reefs, are globally important nesting havens for tropical seabirds (*1*), and provide important habitat for unique island floras and faunas (*2*). However, seabird populations are severely depleted on most atolls, due to a combination of drivers, including introduced predators such as rats that feed on seabird eggs and chicks, conversion of roosting habitat, diseases, and interaction with fisheries (*3*). Similarly, native vegetation on atolls has widely been converted to crops or removed for development, with coconut plantations estimated to now dominate 60% of Pacific atoll forests (*4*). Rat eradication and re-establishment of native forests are thus powerful tools for atoll ecological restoration aimed at biodiversity recovery (*5*, *6*), yet a timely and urgent unanswered question is whether, and to what extent, seabirds and native vegetation can promote key ecological processes that support atoll island climate resilience (*7*–*9*).

Ecological processes can influence atoll island responses to environmental change through provision of key geo-ecological functions. These include (i) vertical reef growth (hereafter referred to as reef accretion), which needs to be sustained at rates that relatively closely match sea-level rise to help maintain island shoreline wave exposure within existing parameters (*10*, *11*), (ii) biogenically derived sediment production in the surrounding shallow marine environments, which is important for maintaining or building islands through either shoreline additions or surface elevation contributions during wave-overtopping (*12*, *13*), and (iii) sediment trapping and binding by fine roots of native vegetation cover to limit wave and storm erosion (*14*, *15*). Given the importance of these geo-ecological functions, the future resilience of atoll islands is likely to be sensitive to present-day ecological conditions. These functions act upon landforms that have formed over recent millennia and which exhibit differing physical attributes, including size, shape (circularity and width), elevation, and reef platform width. Such physical attributes will also contribute to future island resilience (*14*, *16*), but given that atoll islands are naturally active morphodynamic biogenic systems (*8*), understanding the contemporary geo-ecological functions operating upon them and how these functions can be enhanced is an important part of the future island resilience challenge.

Key to unlocking systemic insights into whether and how seabirds and native vegetation influence these geo-ecological functions is the application of a measurable, common currency that transcends and connects marine and terrestrial ecosystems and organisms. Ecosystem energetics provides a useful framework to connect various ecological functions, species guilds, and systems under the general currency of energy flow and energy allocation (*17*, *18*). Studying biological energy flows across the air-land-sea interface enables connections to be assessed among seabirds, forests, and coral reef communities and allows evaluation of how changes in energy flow allocations influence key geo-ecological functions that can support the resilience of atoll islands.

Here, we apply an ecosystem energetics approach to unify terrestrial and marine ecology data and determine how core ecosystem processes and geo-ecological functions relevant to atoll island resilience vary across gradients of seabird densities and native vegetation cover (table S1 and fig. S1). Working on 28 islands across the inner Seychelles, Chagos Archipelago, and Teti’aroa Atoll in French Polynesia (Fig. 1), we provide a linked case-study approach of islands with differing extent of coconut plantation versus native forest cover and differing rat invasion status, and thus differing seabird densities (Materials and Methods; Fig. 1A). Using seabird census data, we estimate the energy that seabirds extract from surrounding pelagic environments and subsequently deposit as nitrogen on the islands. Next, we assess the extent to which seabird nutrients and native vegetation influence key components of island and reef ecosystem metabolism. We demonstrate how gradients in energetics scale with key ecological processes performed by plants, corals, and parrotfish. Last, to inform potential island restoration interventions, we contrast islands with and without rats, and above and below a native vegetation cover threshold to assess how these restoration interventions may influence reef accretion potential, sediment production, and sediment stabilization.

**Fig. 1.**
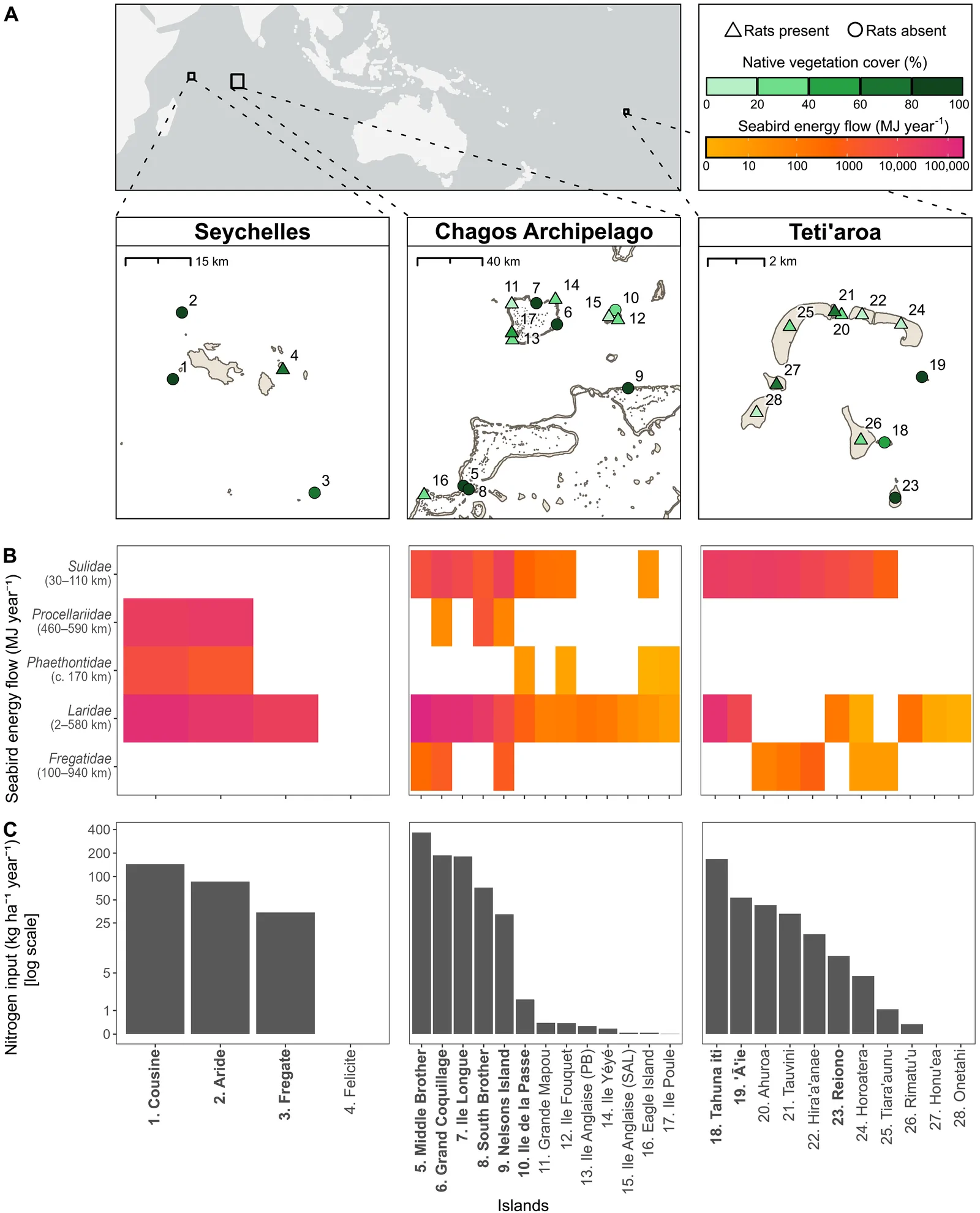
Study locations, seabird energy flow, and nitrogen input. (**A**) The three study locations are spread across the tropical Indo-Pacific, with study islands within each location (Seychelles, *n* = 4; Chagos Archipelago, *n* = 13; Teti’aroa, *n* = 11) varying in their rat status (symbols) and proportion of native vegetation cover (green color ramp). Marine sampling locations are directly adjacent to the study islands. Land and water colors are inverted in the site maps (gray and white, respectively) to maximize visual contrast. (**B**) Seabird population total energy requirements (orange color ramp) from the pelagic environment depicted by seabird family and study island. Seabird family foraging range given below family name. (**C**) Estimated nitrogen input by seabirds per island, with rat-free island names given in bold. Reiono, an island of Teti’aroa, had invasive rats eradicated in 2018.

## RESULTS AND DISCUSSION

We frame the following analyses through an ecosystem energetics perspective, treating energy flow as a common currency linking seabird nutrient inputs, terrestrial fine root productivity, and coral primary productivity and parrotfish energetics.

### Seabird energetics, nutrient subsidies, and native vegetation

We estimated the total seabird energy flow to each island based on species-specific composition and population sizes (fig. S2), to infer the amount of energy seabirds transfer from the surrounding pelagic environment (foraging range up to 1000 km) to the relatively small islands where they breed (Fig. 1B). Within each study location, there was a wide range in seabird energy flow at individual island scales due to differences in seabird compositions and population sizes (0 to 119,427 MJ year^−1^ in Seychelles, 0.87 to 105,884 in Teti’aroa, and 8.37 to 204,387 in the Chagos Archipelago). These differences in seabird communities and energy flow resulted in a large variation of estimated nitrogen input per island (0 to 145 kg ha^−1^ year^−1^ in Seychelles, 0 to 168 in Teti’aroa, and 0.01 to 366 in the Chagos Archipelago; Fig. 1C). The differences in nitrogen input among islands were strongly influenced by rat presence and, to a certain extent, by the proportion of native vegetation [rat × native vegetation interaction estimate = 1.04; 95% highest posterior density interval (HPDI) = 0.98 to 1.09; posterior probability of positive effect (PP) = 0.92], with the highest seabird nitrogen inputs on rat-free islands with high proportions of native vegetation, consistent with previous studies (*19*). Native vegetation cover varied from 4.5 to 100% across the study islands, with abandoned coconut plantations dominating some island interiors. While coconut palms disperse and colonize island perimeters naturally, their wide geographic expansion and monodominance over island interiors has been aided by people, and coconut forests in island interiors are typically abandoned 19th/20th-century plantations that replaced native broadleaf forests and other natural vegetation (*20*).

### Island and coral reef energetics

Focusing on below-ground plant productivity as a key biogenic parameter related to enhancing island sediment stability (*21*), fine root net primary productivity (NPP) rose with increasing native vegetation cover, but the strength of this effect varied with seabird-enriched soil δ^15^N from root core soils (Fig. 2A; native vegetation × soil δ^15^N interaction estimate = 1.01, 95% HPDI = 1.00 to 1.02, PP = 0.98). The estimated nitrogen input of seabirds to each island scaled strongly with soil nitrogen stable isotope ratios, suggesting that these ratios capture nutrient integration into the island ecology (fig. S3A). With abundant seabirds and associated high soil δ^15^N values, root productivity increased by 8.92% for each 1% increase in native vegetation cover (95% HPDI = 3.72 to 16.24), compared to no increase when there were few seabirds present and low soil δ^15^N values (estimate = 0.03%, 95% HPDI = −5.75 to 5.44). The strongest effect on root productivity was native vegetation cover (centered and scaled predictors effect size = 0.61), followed by the interaction of native vegetation cover and δ^15^N (0.44), with the weakest effect for soil δ^15^N (0.12). Native broadleaf trees and vegetation on atolls, particularly *Pisonia grandis*, are typically fast-growing, with long coarse and fine roots due to the high disturbance frequency and distinct resource conditions typical of the environment. In contrast, the coconut palm *Cocos nucifera* has much shorter and rounded root systems. While the influence of seabird nutrients on above-ground plant productivity has been shown, for example, on subarctic islands (*22*), this is the first study to show enhanced root productivity on atoll islands.

**Fig. 2.**
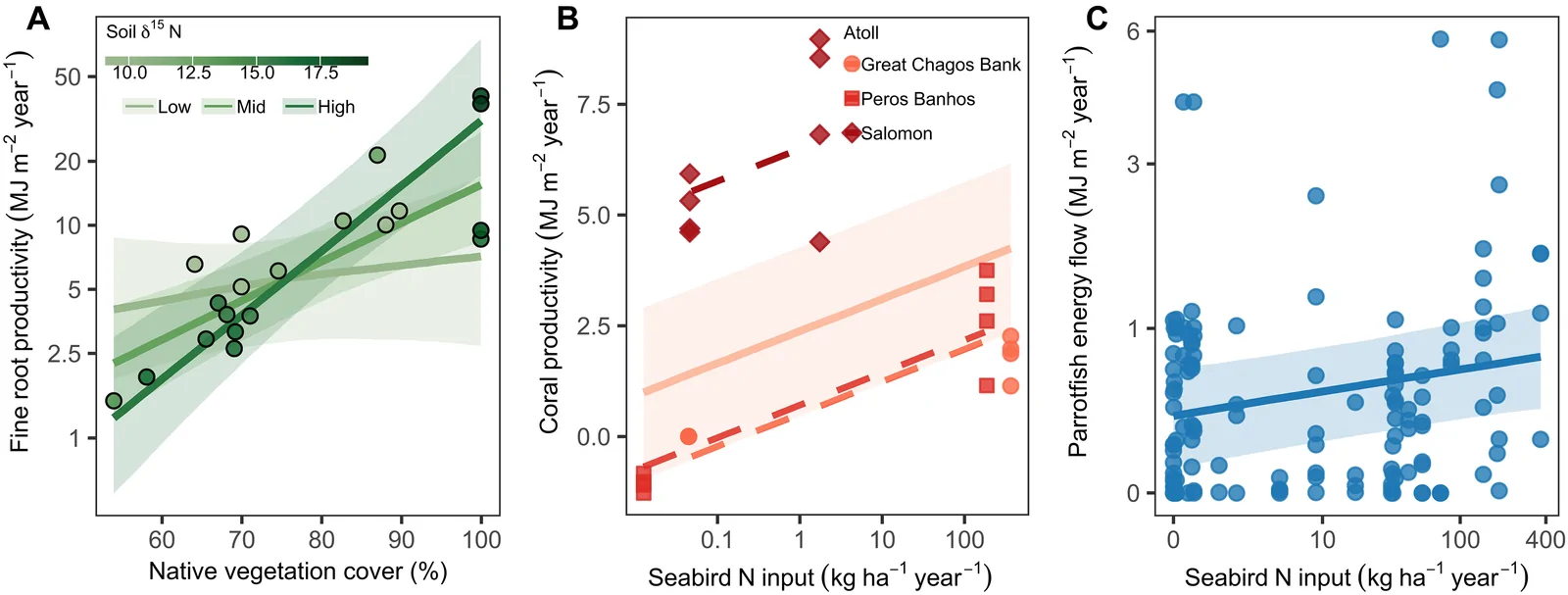
Primary productivity and animal energy flow on islands and surrounding coral reefs in relation to seabird nutrient inputs and native vegetation cover. (**A**) Relationship between native vegetation cover and fine root productivity across three representative soil δ^15^N values indicative of differing seabird nutrient inputs (low = mean – 1 SD, mid = mean, high = mean + 1 SD) with 75% Bayesian credible intervals (*n* = 20 plots across five islands in Teti’aroa, colored by gradient in soil δ^15^N). (**B**) Relationship between seabird nitrogen input to islands and coral community primary productivity on adjacent coral reefs, with 75% Bayesian credible intervals. Solid line = global trend, dotted lines = atoll-level trends due to differing intercepts (*n* = 24 transects across six islands in the Chagos Archipelago). (**C**) Relationship between seabird nitrogen input to islands and parrotfish energy, with 75% Bayesian credible intervals (*n* = 134 transects across 27 islands in all three study locations). All data are analyzed using Bayesian hierarchical models.

Building on previous evidence of seabird nutrients leaching from islands into coral reef food webs (*23*, *24*) and evidence of estimated seabird nutrient inputs scaling with macroalgal nitrogen stable isotope values across our study islands (fig. S3B), we investigated whether seabird nutrients on islands influence primary productivity and energetics of reef-associated organisms. Because coral reef accretion, which contributes to island shoreline protection, is strongly influenced by coral cover and calcification rates (*25*), we quantified the influence of seabird nitrogen input to islands on adjacent coral primary productivity rates. We assessed coral community primary productivity using data on island-specific photosynthesis and respiration rates for *Acropora* spp. and coral cover (Materials and Methods). We found that for each 10-fold increase in seabird nitrogen input to islands, mean coral community primary productivity increased by 0.73 MJ m^−2^ year^−1^ (Fig. 2B; 95% HPDI = 0.47 to 1.00, PP > 0.99). The mechanism driving this relationship is supported by seabird nitrogen input scaling similarly with measured *Acropora* coral net photosynthesis (fig. S4A), meaning that coral cover alone was not driving the pattern. While anthropogenic nutrient inputs can drive negative outcomes for corals (*26*), natural nutrient inputs that are often rich in both nitrogen and phosphorus, such as seabird guano (*27*), can enhance photosynthetic efficiency and result in increased coral calcification (*28*, *29*). For consumer energetics, we focused on parrotfish (Scarinae) as key bioeroders on coral reefs and resultant producers of reef sediment (*30*). For each 10-fold increase in seabird nitrogen input, median parrotfish energy flow increased by 10.3% (Fig. 2C; 95% HPDI = 1.39 to 20.30, PP = 0.99). Seabird nutrients also enhance fish growth rates on surrounding coral reefs (*23*, *31*), which is not accounted for here, so the relationships we provide are conservative.

### Energetics scale with key ecosystem processes

Next, we assessed how differences in energy flow through key components across the land-sea interface scale with processes that can contribute to protecting, building, and maintaining reef islands: coral carbonate production, parrotfish-mediated conversion of reef carbonate to sediment through bioerosion, and root-mediated stabilization of island sediments. A strong positive relationship is expected because variables are not fully independent; what is of interest here is quantification of the scaling relationships between energetics and ecosystem process.

Coral gross carbonate production rates (Fig. 3A), a key variable controlling tropical coral reef accretion (*32*), scaled strongly with coral community primary productivity (with a similar trend apparent if only focused on *Acropora* corals, supporting the extrapolation to the coral community; fig. S4B). For each one-unit increase in coral community primary productivity (MJ m^−2^ year^−1^), coral carbonate production increased by 1.50 kg m^−2^ year^−1^ (95% HPDI = 1.08 to 1.91). Corals gain energy from photosynthetic byproducts of their algal symbionts (zooxanthellae) and through heterotrophic feeding on zooplankton. Where inorganic nutrient availability is high, corals can also digest excess zooxanthellae, which provides a further link between symbiont primary productivity and coral growth (*33*). There is thus a mechanistic link between nutrient-enhanced primary productivity and faster coral calcification and gross carbonate production rates, shown here. This further explains how coral cover rebounds more rapidly after climate disturbance in the presence of seabird nutrients (*24*).

**Fig. 3.**
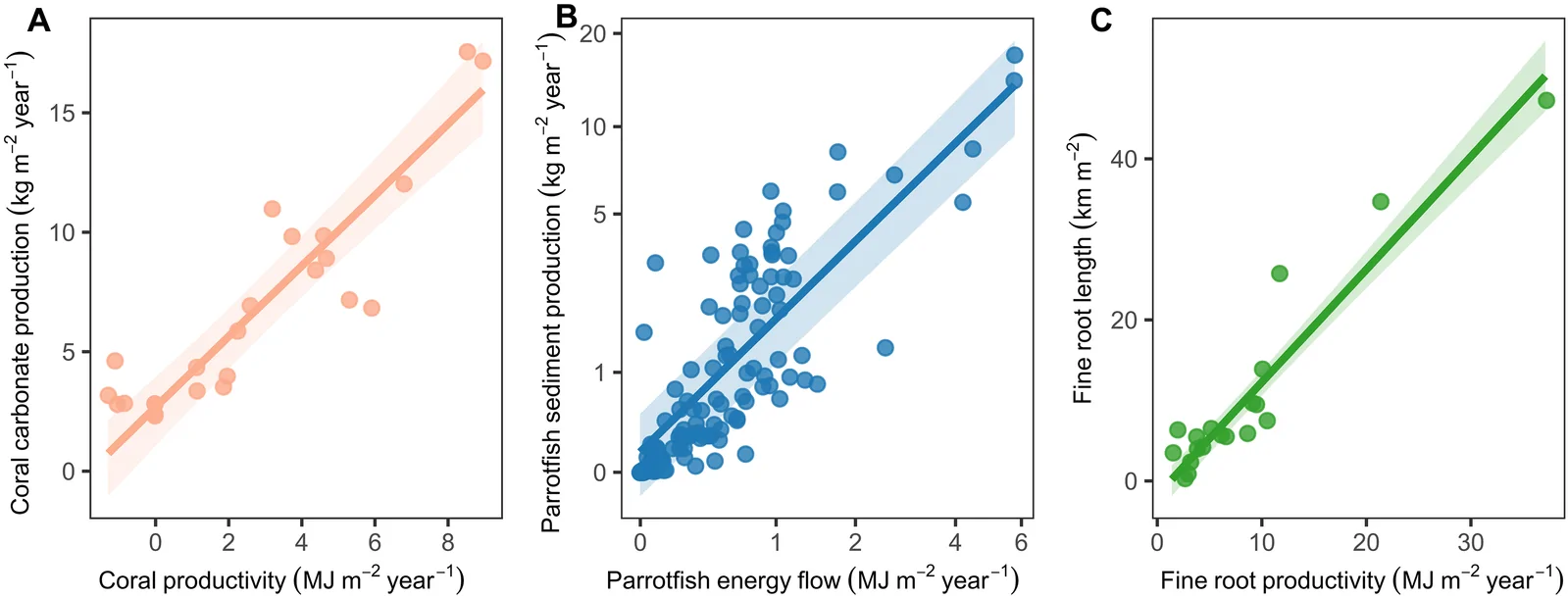
Scaling of primary productivity and energy flow with key ecological processes on islands and adjacent coral reefs. (**A**) Coral community primary productivity calculated from *Acropora* net photosynthesis rates and coral cover against coral carbonate production calculated from colony-level calcification rates (*n* = 24 transects across six islands in the Chagos Archipelago). (**B**) Relationship between parrotfish energy flow and parrotfish sediment generation (*n* = 134 transects across 27 islands in all three study locations). (**C**) Relationship between fine root NPP and fine root length (*n* = 19 plots across five islands in Teti’aroa). All data are analyzed using Bayesian hierarchical models, with 75% Bayesian credible intervals around trends.

Parrotfish have been estimated to generate up to 85% of new sand-grade sediments on coral reefs in Indian Ocean locations (*30*). Parrotfish-mediated sediment production rates scaled steeply with the metabolic energy flow through parrotfish (Fig. 3B), whereby for each doubling of energy flux through parrotfish, parrotfish sediment production increased by a factor of 2.51 (95% HPDI = 2.25 to 2.80). As parrotfish sediment production is strongly influenced by species-specific abundance and size (*34*, *35*), and seabird nutrient input increases parrotfish growth by ∼35% (*31*), seabirds have the potential to substantially enhance bioerosion and associated sediment production.

Wave processes transport sediment onto islands, and once deposited, plant fine roots help to bind and stabilize this sediment. Total fine root length does not necessarily scale with root biomass production rates, as it also depends on mean root lifetime and variations in root morphology (*36*). Here, we found a tight relationship between root productivity and length (Fig. 3C); for one-unit increase in root productivity (MJ m^−2^ year^−1^), fine root length increased by an estimated 1.41 km m^−2^ (95% HPDI = 1.15 to 1.67). Mean root lifetime was consistently about 2 years, more typical of a tropical montane forest than a lowland tropical forest (*36*), likely because of the poor nutrient and mineral quality of the sandy soils found on atoll islands. Root lifetime did not decrease in more productive forests despite the likelihood that the roots adopt more acquisitive traits.

Given these tight relationships, there were also direct relationships between seabird nitrogen input and native vegetation cover with the three ecological processes (fig. S5). Taking an ecosystem energetics lens provides a common framework to quantify how the influence of seabirds and native vegetation propagates through the atoll system, enabling an uplift in key geo-ecological processes.

### Ecosystem restoration to increase island resilience

The data above demonstrate the positive influence of increasing native forest cover and seabird nutrient inputs on energy flows across the land-sea interface, and how key processes (i.e., coral carbonate production, parrotfish sediment generation, and fine root length) that underpin geo-ecological functions scale with energetics. Next, we tested whether the overall geo-ecological functions of vertical reef accretion potential, reef-wide sediment production potential, and soil binding potential (approximated through relative increases in fine root length per unit area) are elevated under key preservation or restoration scenarios. Specifically, rat eradication and native revegetation are active restoration approaches for atoll islands (*5*, *6*), aimed at mitigating two of the most pervasive threats to atoll ecology across the Indo-Pacific, and provide potential intervention levers to influence the nature-based ecosystem dynamics we document here. To test how effectively these ecologically mediated controlling factors could enhance the physical resilience of atoll islands, we focused on the low-lying atoll islands of the Chagos Archipelago and Teti’aroa (Materials and Methods). We assessed overall reef accretion rates (mm year^−1^) and sediment production potential (m^3^ m^−1^ shoreline year^−1^) on atoll islands in the Chagos Archipelago with and without invasive rats (Materials and Methods), indicative of seabird presence, with rat eradication representing a primary island conservation strategy (*37*). We also contrasted fine root length estimates at sites in Teti’aroa below and above a 75% native vegetation threshold, which previously has been used as a native vegetation restoration target for atoll islands (*19*), and native vegetation being the strongest driver we found of fine root productivity. Native vegetation was similar between Teti’aroa and Chagos Archipelago sites, with *P. grandis*, *Calophyllum inophyllum*, and *Guettarda speciosa* all common, as they are across almost all atolls in the Indo-Pacific.

Reef accretion potential, which includes gross carbonate production by corals and crustose coralline algae (CCA) and a proportion of sediment production that infills accumulating framework (Materials and Methods), was highest on lagoonal reefs around rat-free islands (that host large seabird colonies; median birds ha^−1^ = 2895), where median reef accretion was estimated to be 5.92 mm year^−1^ (95% HPDI = 2.93 to 8.75). This was 2.64 mm year^−1^ (1.8 times) higher than on lagoonal reefs around rat-infested islands (hosting few seabirds; median birds ha^−1^ = 0.50) (Fig. 4A; 95% HPDI = 0.65 to 4.70, positive rat-free effect PP = 0.99). There was also higher reef accretion potential on shallow ocean-facing reefs around rat-free (median = 3.58 mm year^−1^, 95% HPDI = 0.55 to 6.30) versus rat-infested (median = 1.42 mm year^−1^, 95% HPDI = −1.42 to 4.37) islands (Fig. 4C; 95% HPDI = 0.09 to 4.16, positive rat-free effect PP = 0.98). The difference in reef accretion potential, especially on lagoonal reefs, is meaningful in the context of projected rates of future sea-level rise. Specifically, accretion rates calculated for rat-free lagoonal reefs (∼5.9 mm year^−1^) would be sufficient for reefs to closely track predicted sea-level rise rates through to 2100 under a 2°C warming scenario [∼6 mm year^−1^ for the study location; (*38*)]. If these rates are realized, this could help mitigate wave-driven flooding magnitudes (*11*) and reduce lateral erosion of island coastlines. Conversely, lagoonal reefs adjacent to rat-infested islands are predicted to have limited capacity to keep pace with even current rates of sea-level rise in the study region (∼3.3 mm year^−1^). Lagoonal reefs in many atolls receive considerable wave energy, particularly for large or semi-open atolls, and so the gains calculated around rat-free islands are of considerable importance. The overall lower reef accretion values on outer ocean-facing reef settings reflect the often wide and sparsely colonized reef pavement habitats that typify these more exposed environments. However, even in these more dynamic settings, the additional carbonate material produced next to rat-free islands has the capacity to be either retained in the reef to drive in situ accretion, or to be transported landward to contribute to island-fringing rubble flats and storm ridges (*39*).

**Fig. 4.**
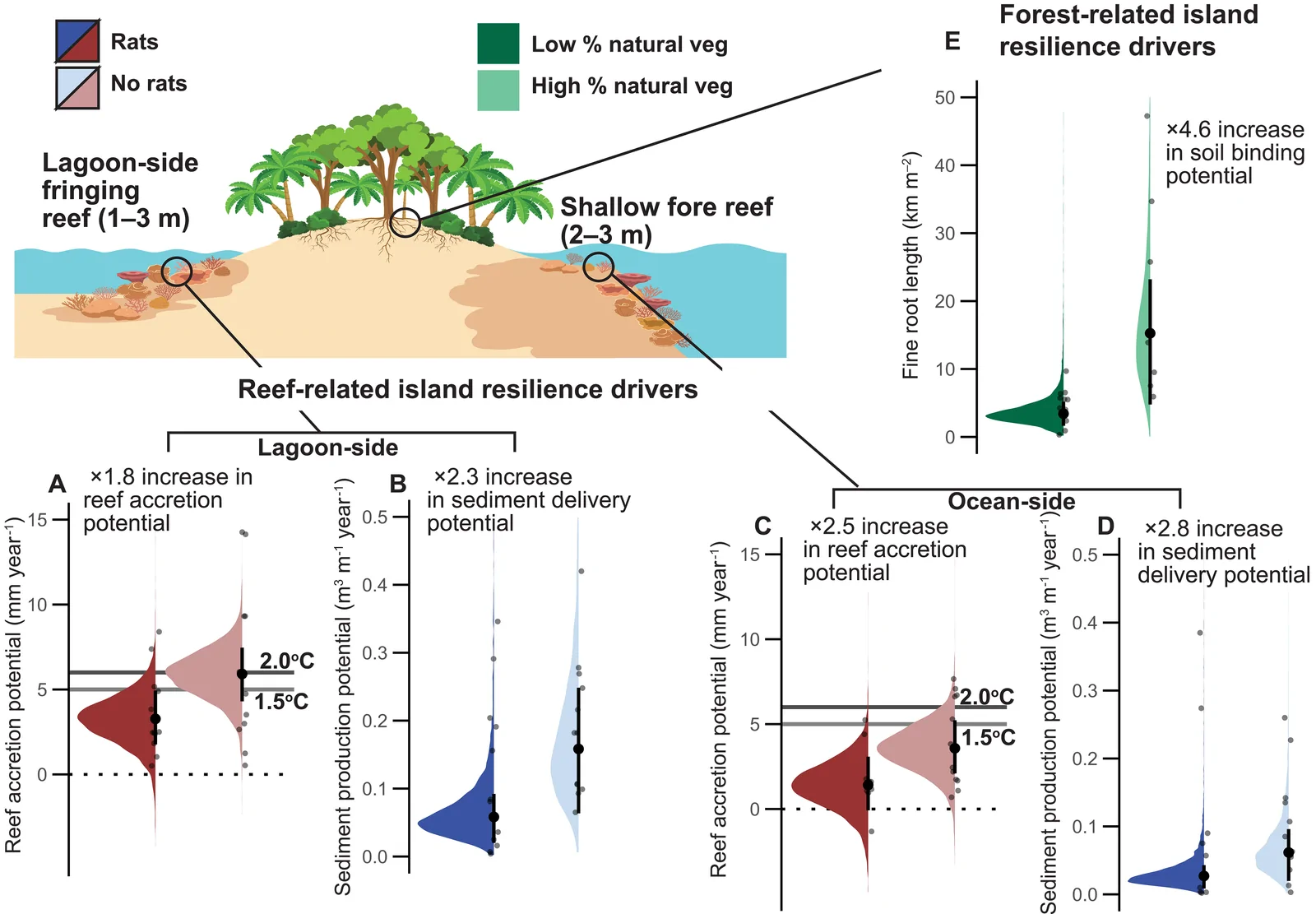
Geo-ecological functions enhanced by seabirds and native vegetation. Reef accretion potential (RAPmax) on (**A**) lagoonal and (**C**) forereef sites adjacent to islands with and without invasive rats (*n* = 48 transects across six islands in the Chagos Archipelago). Horizontal gray lines denote projected rate of sea-level rise (mm year^−1^) for the central Indian Ocean in 2100 under 1.5° and 2.0°C warming scenarios. Values of constituent components of reef accretion potential per site are available in table S2. Sediment production potential on (**B**) lagoonal and (**D**) forereef sites adjacent to islands with and without invasive rats (*n* = 47 transects across six islands in the Chagos Archipelago). (**E**) Fine root length measured in forest plots (*n* = 19 across five islands in Teti’aroa) above and below a 75% native vegetation cover threshold (split also by rat status in table S3). Half-eye plots depict the Bayesian posterior distributions, the median (large point), 75% credible intervals (lines around the medians), and raw data points (small points). All data are analyzed using Bayesian hierarchical models.

Sediment production potential, which includes quantification of sediments produced by parrotfish, sea urchins, *Halimeda*, foraminifera, and molluscs, was higher for rat-free islands than rat-infested islands, with estimated 2.3 and 2.8 times higher median sediment production rates (and potential delivery to islands) on lagoonal reefs (0.16 versus 0.06 m^3^ m^−1^ shoreline year^−1^) and shallow outer forereefs (0.06 versus 0.03 m^3^ m^−1^ shoreline year^−1^), respectively (Fig. 4, B and D; lagoon: 95% HPDI = 0.62 to 7.05, positive rat-free effect PP = 0.97; forereef: 95% HPDI = 0.50 to 5.88, positive rat-free effect PP = 0.93). This estimated uptick in sediment production around rat-free islands is substantial. For example, for a hypothetical island of ∼14 ha (1200 m long by 150 m wide) and with reefs 1000 m long and 200 and 150 m wide, respectively extending along lagoon and seaward shores, we estimate that an additional ∼168 m^3^ of sediment would be produced per year around rat-free compared to rat-infested islands. While not all of this sediment will reach the island, there is a high degree of consistency in sediment type and grain size between on-reef sediment production estimates, and beach sediment deposition in this reef system (*35*). This is suggestive of a strong sedimentary linkage between the reefs and islands. While the strength of this linkage will vary among island contexts, coral sand and rubble are important contributors to island materials across many Pacific, Indian, and Caribbean islands (*40*), suggesting that seabird nutrient enhanced coral growth and sediment production from bioerosion of coral substrate may be of importance to many other reef islands.

Fine root length per unit area was an estimated 4.6 times higher (95% HPDI = 0.02 to 20.7, native vegetation PP = 0.96) in areas of high native vegetation compared to areas of low native vegetation, substantially increasing soil binding potential (Fig. 4E). For sites with over 75% native vegetation, estimated median fine root length was a remarkable 15.8 km m^−2^ (95% HPDI = 0.07 to 49.52), while for sites under 75% native cover, the median estimate was 3.42 km m^−2^ (95% HPDI = 0.19 to 8.02). While soil depth on atoll islands is shallow, typically giving way to larger sediments and carbonate material with increasing depth, vegetation stabilizing this soil lens appears important, particularly during island wash over events. Compared to coconut plantation cover, natural vegetation cover can substantially reduce island erosion and island area loss following severe storms when waves wash over the island and can scrape off interior soil (*41*, *42*). Furthermore, island vegetation density is one of the most important variables in reducing long-term wave-driven erosion (*14*). A large part of the ability of native vegetation to reduce long-term and storm-driven island erosion is likely due to the higher fine root length we report here, which stabilizes and binds soils (*43*).

### Study wider implications

Collectively, our findings across these case study islands provide insights into how contemporary terrestrial and marine processes, enhanced by high seabird densities and high native vegetation cover, may help support the resilience of low-lying atoll islands. While additional contextual factors, such as island size or enabling oceanographic conditions, also influence reef island robustness to climate change threats, the three geo-ecological functions we explore here are important contributing factors that may limit island vulnerability and promote island growth and stability (*10*, *15*). We demonstrate how increased seabird presence enhances an island-coral reef feedback loop, boosting the geo-ecological functions that support the stability of the very islands on which they breed and roost. This feedback highlights how biological connectivity across air, land, and sea can generate self-reinforcing resilience in atoll systems.

Our assessment of functional gains is focused on sediment-built atoll islands, common to the central Indian Ocean, whereas many islands, especially in the Pacific Ocean (*16*), are composed of larger carbonate material (especially coral rubble). However, as much of this larger material is also coral derived (*40*), it is possible that seabirds also influence these other island types, for example, through increased coral growth and enhanced event-driven coral rubble generation. Seabird-derived guano coupled with *Pisonia* native interior forests can also lead to island-consolidating phosphate rock formation, further stabilizing islands (*44*). While climate warming is a major threat to coral cover and associated reef accretion rates (*25*), seabird nutrients have been shown to increase coral growth rates by two to four times across multiple species (*24*, *45*, *46*) and substantially shorten the time needed for coral cover to recover after bleaching events (*24*). These more resilient reefs are thus likely to sustain the reef accretion rates we document here under moderate climate change scenarios.

Our findings quantify nature-based solutions aimed at bolstering key processes that enhance atoll resilience (*9*) and therefore support island restoration efforts. Removing invasive predators, particularly rats, from islands is a burgeoning island restoration strategy. For instance, rats have been removed from ∼1000 islands to date, at an escalating rate and scale driven by increasingly ambitious island restoration targets (*5*). While such interventions can be costly, increasing with island size and remoteness (*47*), eradications are becoming more efficient through the development of new bait deployment methodologies (*48*). The removal of abandoned coconut plantations and restoration of natural vegetation are also restoration interventions that are gaining traction and may need to be done in conjunction with rat eradications to support seabird returns (*6*, *49*). Restoration success will likely be expedited and enhanced through social attraction and/or translocation approaches to speed up recovery of seabird populations (*50*).

While we took a space-for-time approach, the relevance to restoration efforts is supported by an extensive literature on conservation benefits following invasive mammal eradications (*37*), experimental manipulations demonstrating the integration and influence of seabird nutrients on marine organisms (*24*), and evidence of seabird nutrient subsidies developing from islands following rat eradication (*51*). As such, in combination with fisheries management to maintain parrotfish populations, our findings provide evidence that island restoration approaches are not only tools for biodiversity-centered conservation, but can enhance the resilience of low-lying atoll islands, particularly the many sparsely populated, agricultural, and uninhabited islands [which make up 93%, or ∼7250, of Indo-Pacific atoll islands; (*52*)], for which engineering solutions are unfeasible, inappropriate, and costly. To date, ecosystem restoration on atoll islands has been primarily motivated and funded by species conservation ambitions, but our study providing a mechanistic link between atoll biodiversity and climate resilience may open pathways to use climate-adaptation funds for rewilding atolls as a resilience-building tool. Funding of island restoration across atolls will require innovative conservation finance approaches, including partnerships between governments, NGOs, donors, and international adaptation funds.

While the increases in key geo-ecological functions we report here seem likely to support island resilience, challenges exist in explicitly linking these contemporary functions to long-term island physical attributes and temporal dynamics. Complex interdisciplinary research will be needed to quantify and model, for example, sediment entrainment and transport rates from reefs to islands and where these processes are maximized. Similarly, a more in-depth understanding of the links between reef accretion, wave energy, and lateral island shoreline scouring needs to be developed, along with the role of native vegetation fine roots in preventing erosion across different island contexts. These constraints aside, atoll island systems provide a model for exploring how multiple ecological and geo-ecological processes across marine and terrestrial ecosystems (seabird nutrient transfer, vegetation root production, coral reef accretion, and sediment production) interact to influence landforms. Our results indicate how rewilding such interconnected ecosystem processes may increase resilience to the impacts of climate change. Such insights on the utility of restoration for reinforcing resilience are likely to apply to many other ecosystems but may not be as straightforward to isolate. Atolls provide an excellent system to describe such emergent resilience.

## MATERIALS AND METHODS

### Study sites

We studied 28 islands across three geographies (inner Seychelles, Chagos Archipelago, and Teti’aroa Atoll, French Polynesia) in the Indo-Pacific region. All islands were either uninhabited or very sparsely inhabited by a small research station or ecotourism center, with no or negligible fishing pressure. The islands were chosen to represent differences in rat presence, seabird population densities, and native vegetation cover. No other invasive mammals were present on any of the islands. The four inner Seychelles islands are high granitic islands used to help build understanding of links between energetics and processes across tropical systems more generally, but not the geo-ecological functions supporting atoll island resilience. The other 24 islands are all low-lying atoll islands. Islands varied in rat status, seabird densities, and native forest cover within all three locations. Rat presence was a weaker predictor of seabird densities at Teti’aroa because some islands had rats removed in the 5 years prior to our field sampling, and because some coastal habitats can support red-footed booby colonies despite rat presence. The dominant human-altered forest cover across the study islands was abandoned coconut plantations representing historic land-use history, defined as near-monodominant coconut vegetation in the interior of an island, away from the coastal island fringe where they occur naturally. Percent native vegetation cover was estimated using satellite imagery and island mapping to estimate the proportion of each island covered by coconut plantations or human settlement.

Many of the locations we worked at are very remote and logistically challenging, meaning some measurements could only be conducted at some geographies. For these reasons, we took the strategic decision to focus on collecting the best data we could for each relevant parameter at the location or locations most suited to the overall research question (table S1). Some measurements are directly measured from survey data or chemical analyses, while others combine survey data with modeling from published parameters or are inferred from taxa specific rate data (table S1). In the Seychelles, we installed nine forest plots across four islands (November 2022 to November 2024). Marine surveys of fish communities were conducted at eight sites across the four islands. In the Chagos Archipelago, we installed 15 forest plots on nine islands, conducted surveys of fish communities at 13 islands, and detailed marine geo-ecological surveys of reef carbonate production and sediment production at six islands. In Teti’aroa, French Polynesia, we installed or recensured nine forest plots across nine islands, with five NPP plots monitored over a 2-year period (August 2022 to August 2024). Surveys of fish communities were conducted at 13 sites across 10 islands. Soil samples were collected from forest plots (*n* = 5 samples per plot) and macroalgal samples from marine survey sites (*n* = 6 to 12 per site) for nitrogen stable isotope analysis across all study locations. Seabird censuses were conducted across all 28 study islands. Differences in sample sizes across different surveyed components were due to logistics of access for repeat sampling, and a focus on low-lying atoll islands for geo-ecological functions.

### Field data collection

#### Seabird densities

Breeding seabird densities were estimated by counting the number of apparently occupied nests (AONs) on each island in the peak breeding seasons. For seabirds in the Seychelles, annual census data are collected in the peak breeding season during the southeast monsoon. We used census data from 2021 to 2023 to match our island and reef surveys. For Teti’aroa, we surveyed seabird densities during the peak breeding season (October to November) in 2021 to align with most of our island and marine surveys. In the Chagos Archipelago, seabird surveys were conducted in different months between 2008 and 2018, and we used data from peak breading months in this period to match peak values used from other locations (*53*). Sampling regimes varied due to logistical constraints and differing environmental conditions that restricted access to certain sites at certain times leading to variable time in the field. In all sites, counts of AON were initially conducted around the coastline of each island. Counts were then conducted within the islands’ interiors for large species (i.e., *Sula* spp. and *Fregata* spp.) and ground-nesting terns (e.g., *Thalasseus bergii*). For smaller species (e.g., *Anous* sp.), burrowing species (e.g., *Puffinus* sp. and *Ardenna* sp.), and sooty terns (*Onychoprion fuscatus*), counts of nests and occupied burrows were conducted within random plots that were considered representative of each island or along transects (using visual or acoustic playback methods) and were then extrapolated to the islands’ interior surface areas or to the extent of the suitable habitat where this was known. For tropicbirds (*Phaethon* sp.), aerial displaying pairs were counted.

#### Forest plots (including NPP)

Forest plots (see sample sizes above) were established and censused according to the protocols of the pantropical Global Ecosystems Monitoring (GEM) Intensive Vegetation Plot network (*54*). Plots were placed in representative habitats on each island. GPS coordinates of plot corners and each tree location were taken using a differential GPS (Emlid RS+), with plot area varying from 20 m by 40 m to 100 m by 100 m depending on vegetation diversity (larger plots for greater diversity sites). All censuses are accessible on ForestPlots.net. Soil ^15^N samples were used from all plots, while NPP and fine root length were calculated for five plots in Teti’aroa, where regular repeat sampling of canopy and root productivity was possible. Data were collected over a 24-month period from 2022 to 2024. More information is given in the forest primary productivity section below.

#### Marine surveys

Fish community surveys were conducted across all three geographic locations (Seychelles in 2022, Chagos Archipelago in 2021 and 2023, and Teti’aroa in 2021). All surveys were conducted along four 30-m transects per site (except for one site in Teti’aroa where we could only fit two transects), which were randomly positioned along shallow depth contours (3 to 6 m depth in Seychelles, <3 m depth in the Chagos Archipelago, and <2 m depth in Teti’aroa) to be offshore from forest plots on the islands. All noncryptic, diurnal, reef-associated fish were surveyed at species level, recording abundance and body sizes along each replicate transect. Larger, more mobile fish species, including the parrotfishes of interest in this study, were surveyed within 30 m–by–5 m belt transects, while small site-attached Pomacentridae were surveyed within 30 m–by–2 m belt transects. All surveys were conducted by experienced fish surveyors (N.A.J.G. and C.E.B.). Surveys were conducted on lagoonal reefs in the Chagos Archipelago and Teti’aroa, while sites in the Seychelles were located on one or several sides of islands as there is no predominantly sheltered aspect. Detailed measurements of coral colony sizes and other calcifiers as well as sediment producers were conducted at six islands across the Chagos Archipelago at both lagoonal (1 to 3 m depth) and shallow fore reefs (2 to 4 m depth) to enable carbonate and sediment production estimates. Surveys were conducted along four 10-m transects following the three-dimensional (3D) reef contour to calculate reef framework production and sediment production (see the “Processes and functions” section).

#### Isotope samples and analysis

Five soil samples were collected for isotope analyses from topsoil (0 to 10 cm) in all forest plots. Six to 12 locally abundant macroalgal samples were taken from each marine survey site. Samples were dried until fully dry. They were homogenized and ground, prior to weighing. Samples were run for δ^15^N at the isotope facilities at Lancaster University, UK, and University of Texas, USA. Accuracy based on international standards was 0.2 per mil standard deviation, with selected samples run in duplicate or triplicate to ensure precision of readings.

### Energetics

#### Seabird energetics and nutrient input

We compiled data on the average reported maximum foraging ranges of the different seabird species from the Seychelles, Chagos Archipelago, and Teti’aroa using expert knowledge and published literature. To estimate the spatial distribution of the seabird populations’ energy requirements during the breeding season, we first adjusted our numbers of AONs to estimate the total number of birds on each island, using a conservative estimate of three birds per nest (*23*). We then estimated daily species-specific breeding field metabolic rate (FMR) using species-specific adult body mass data (*55*), breeding colony latitude, and the phylogenetically informed model by Dunn *et al.* (*56*). To calculate the total energy expenditure of each population over their breeding season, we followed the methods of Graham *et al.* (*23*) and multiplied this value of FMR by the number of days that the species generally spends at the breeding colony each year (*53*) and the proportion of the day that an individual spends there (*55*). To convert these energetic expenditure values into the total energy requirements of each seabird population, we divided energy expenditure by an assimilation rate of 75% (*57*). We estimated the quantity of nitrogen that seabirds input into the environment over the breeding period based on species-specific defecation rates based on assumed allometric scaling (*58*) and mean guano nitrogen content, derived from guano samples collected in the Chagos Archipelago and the Seychelles (mean = 9.6%, range = 1.1 to 34%, *n* = 295).

#### Forest primary productivity

Forest NPP was assessed across five forest plots in Teti’aroa using the procedures of the GEM protocol (*54*). Fine root production was measured using wire root cores (15 cm deep and 12 cm in diameter) inserted into the ground at each corner of the plot (*n* = 4). Root cores were placed in open areas, avoiding direct proximity to trees. At installation, all roots were extracted, and the core was filled with the root-free soil. The root mass at the first installation quantified root biomass rather than production. This first root harvest was analyzed for carbon concentration during the isotopic analysis described above. The cores were then harvested every 3 months, cleaned, dried (70°C until constant mass), and weighed. Roots in each core were extracted for a fixed amount of time (3 × 10 min), and the total root mass in the sample was estimated from a cumulative root mass over time curve (*59*, *60*) solved to 120 min. This method allows the root search time to be kept within a reasonable limit in the field while taking into account that a small fraction of roots is likely to remain unextracted. Root NPP measurements were averaged over 1 year and scaled to 1 m^2^, to provide a standard unit of measurement (Mg C m^−2^ year^−1^). Root NPP was converted to energy (MJ m^−2^ year^−1^) by multiplying NPP by 39.6 kJ g^−1^, the calorific content of dry root matter.

#### Coral NPP

We measured light photosynthesis and dark respiration of *Acropora* spp. across six sites in the Chagos Archipelago (three rat-free and three rat-infested). Five coral fragments were collected at 1.5 m depth at each site. Each coral fragment was incubated in airtight glass jars (i.e., no water flow) on the reef in 1.5 m depth at noon (saturated light) and in a closed coolbox (dark) for 30 ± 5 min. O_2_ concentration was measured before and after incubation with a handheld oxygen probe. Net O_2_ production/respiration (mg liter^−1^) was normalized to incubation time, coral fragment surface area, and incubation seawater volume (volume glass jar − volume coral fragment) before being combined to net daily photosynthesis (assuming 12 hours of light photosynthesis and 12 hours of dark respiration). Surface area and volume of coral fragments were determined using photogrammetry and 3D modeling (*61*). Net photosynthesis rates (in mg cm^−2^ day^−1^) were translated to MJ m^−2^ year^−1^ using molar and energy conversion constants and multiplied by overall coral cover, to generate site-specific NPP rates. The same process was repeated using just *Acropora* coral cover to help validate the relationships and extrapolation to the wider coral community.

#### Reef fish metabolic rates

For each fish recorded in marine surveys, biomass (g) was calculated using the formula mass = *a* × length*^b^*, with *a* and *b* parameters obtained from species-specific Bayesian estimates on FishBase. FMR was calculated as FMR = θ × SMR, where θ is an activity parameter and SMR is standard metabolic rate that equals *B*0 × mass^α^. We used published family-specific estimates for the parrotfishes for θ, *B*0, and α (*62*, *63*). Mean sea surface temperature, which influences metabolic rate, was 28°C at our study sites (*64*, *65*). Following Schiettekatte *et al.* (*66*), we used Parravicini *et al.* (*67*) to assign a diet group (herbivore/detritivore), which was associated with an assimilation efficiency (AE) based on Schiettekatte *et al.* (*66*). Energy consumption (MJ m^−2^ year^−1^) was then calculated by dividing FMR/AE. Although the cost of growth also influences energy consumption (*62*, *63*), we did not include it to match calculations for other taxonomic groups examined here. In addition, we tested the effect of including versus excluding growth on estimates of fish energy consumption using a subset of our data and found that the estimates were highly correlated (0.97).

### Processes and functions

Fine root length was quantified at first installation of the root productivity cores in Teti’aroa, using the fixed-time count method. A representative subset of roots were scanned for length measurement from five randomly selected root cores and weighed to describe the relationship between fine root length and mass [specific root length (SRL)]. The average SRL from the five root cores (842.21 ± 231 cm g^−1^) was applied to the initial mass measurements of each root core to estimate fine root length per unit area (*68*), an indicator of soil binding potential.

Coral carbonate production rates were estimated on lagoonal reefs of six islands in the Chagos Archipelago, using the ReefBudget survey method (*25*) (http://geography.exeter.ac.uk/reefbudget/). Contour lengths of each coral colony (recorded to genera and morphological level, e.g., *Acropora* branching, *Porites* massive, etc.) were measured under a transect tape, with the colony sizes collected as a function of the true 3D surface of the reef, including cover on overhangs and vertical surfaces. Survey data were entered into the ReefBudget spreadsheets, which use the morphology and size of individual coral colonies in combination with genera/morphotype-specific calcification rates to estimate total annual carbonate production (*G* = kg CaCO_3_ m^−2^ year^−1^). We used relevant calcification rates for sites with and without rats to factor for enhanced calcification around islands with high seabird densities (*46*). This is justified based on (i) compelling and mechanistic evidence that coral growth rates are enhanced with seabird nutrients across the Chagos Archipelago (*24*, *33*); (ii) enhanced coral growth rates associated with seabird nutrient inputs have also been demonstrated elsewhere, such as Fiji (*45*); and (iii) these nutrient-driven differences in growth rates and calcification have been demonstrated across multiple coral species (*24*, *33*, *45*, *46*).

Parrotfish sediment production rates were calculated from abundances and sizes of parrotfishes (>10 cm) surveyed on the underwater surveys at all marine survey sites. These data were combined with species-, size-, and treatment-specific erosion rates to estimate annual sediment production rates (kg m^−2^ year^−1^) (*34*, *35*).

For six islands (three rat-infested and three rat-free) in the Chagos Archipelago, additional detailed calcium carbonate framework and sediment generation rates were estimated on both lagoonal and seaward reefs using the survey-based ReefBudget (see above) and SedBudget methodologies (*35*) (http://geography.exeter.ac.uk/sedbudget/). Additional to coral carbonate production detailed above, carbonate framework rates included CCA surveys and region-specific calcification rates. Additional sediment production from groups other than parrotfish was estimated by combining abundances of gastropods, bivalves, sea urchins, foraminifera, and calcifying erect macroalgae (*Halimeda* spp. and *Udotea* sp.) with species- or group-specific carbonate content and turnover rates. Around seabird-rich islands in the Chagos Archipelago, coral calcification rates are two times higher (*46*), and parrotfish erosion rates are 0.8 times lower compared to seabird-free islands, with underpinning calculation metrics in the ReefBudget and SedBudget methodologies adjusted to factor for these differences. Sea urchins were rare and small in the Chagos Archipelago, and average published sea urchin erosion rates were used throughout.

Maximum reef accretion potential (RAPmax) (mm year^−1^) was estimated for lagoon and shallow ocean-facing forereef sites at the six islands in the Chagos Archipelago. Reef accretion estimates are based on the conversion of measured site-specific gross carbonate production (from corals and CCA) and sediment production [by parrotfish (50%), sea urchins, and macrobioerosion], which infills accumulating reef framework (*25*, *69*). To keep our estimates conservative, we worked on the assumption that only ∼50% of this bioerosional sediment yield is actually incorporated back into the reef [based on data in Hubbard *et al.* (*70*)] and excluded any sediment generation by other benthic sediment producers (e.g., *Halimeda*). We also factored for variations in accumulating reef framework structure as a function of coral community type (60% for the *Acropora*-dominated lagoon-side fringing reefs and 50% for mixed assemblages associated with the shallow forereef sites) based on more conservative recent estimates of framework stacking porosity values (*71*). Stacking porosity is the vertical space added for a given volume of carbonate produced by an assemblage of corals and is a key metric used in converting reef carbonate production data to estimates of reef accretion (*25*). Due to the absence of empirical data on rates of physical reef framework removal or chemical dissolution per unit area of reef surface over time, we did not factor in physical loss rates or chemical dissolution of the substrate.

Sediment production potential was estimated per meter shoreline (m^3^ m^−1^ year^−1^) based on the approach described in Ainési *et al.* (*13*). In brief, sediment production rates measured at each of our Chagos Archipelago study sites were translated (assuming a carbonate sediment density of 1.8 g cm^−3^) to the volume (m^3^) of sediment available for potential delivery to island shorelines per unit area of reef. Here, we used an average reef width of 237 ± 29 m on lagoon reefs (58 ± 7% of which is reef structure), and a reef width of 160 ± 38 m on shallow forereefs (76 ± 5% of which is reef structure). Reef flat width and percent area of reef structure were measured around the six survey islands from GoogleEarth images imported to ImageJ, and results coincide well with previously measured lagoonal reef flat width for 12 islands across the Chagos Archipelago (230 ± 55 m) (*23*) and the distance of nutrient impacts across reefs (*51*).

### Statistical analysis

Because we collected data at multiple spatial scales across three locations (Seychelles, Chagos Archipelago, and Teti’aroa), we analyzed all responses using Bayesian hierarchical models. The specific group-level effects varied for each model and are detailed below.

We described island-level seabird energy requirements using a heatmap approach, with island specific seabird nitrogen input estimates given below. We tested the effects of rat status and native vegetation on island-level seabird N input across all study sites, with atoll nested within location as group-level effects.

Terrestrial fine root productivity data were collected in Teti’aroa, so we included island as a group-level effect and tested for an effect of soil δ^15^N, native vegetation cover, and their interaction on these three parameters. Marine energetic responses—coral productivity and fish energetics—were modeled as a function of seabird nitrogen input to islands to reflect nutrient subsidy potential. Data on coral productivity was evaluated at the Chagos Archipelago, so we included atoll as a group-level effect. Fish were surveyed across all three locations, so atolls nested within location were included as group-level effects.

To link energetics with processes, we modeled root length as a function of root productivity, coral carbonate production as a function of coral productivity, and parrotfish sediment production as a function of parrotfish energetic requirements. Marine process responses were focused on the Chagos Archipelago, so we included atoll as a group-level effect.

Last, we examined how different restoration scenarios may influence geo-ecological functions underpinning atoll resilience. We tested how native forest cover [categorical, below 75% cover versus above 75% cover, following Dunn *et al.* (*19*)] influences root biomass, focusing on Teti’aroa atoll, including island as a group-level effect. Marine responses of reef accretion potential and sediment delivery were measured on both lagoonal and shallow forereefs in the Chagos Archipelago, so we tested for an effect of rat status (categorical, present versus absent), reef location (categorical, lagoon versus forereef), and their interaction, with atoll as a group-level effect. We ran a sensitivity analysis to test for island-specific responses, with models including island nested within atoll as hierarchical grouping factors for both reef accretion and sediment production. This produced nearly identical estimates to the models with just atoll as a group-level effect (fig. S6).

All models were run in R and implemented in STAN using the package brms, with weakly informative priors for four chains, with 3000 iterations and a warm-up of 1000 iterations per chain. All continuous predictors were centered. Model convergence and fit were checked using posterior predictive plots, traceplots, and the Gelman-Ruban convergence diagnostic (R-hat).
