# Supplementary material for: Seabirds and native vegetation drive ecosystem processes that support atoll island resilience
Source: Sci Adv. 2026 Oct 14;12(42):eaei1145. doi: 10.1126/sciadv.aei1145 (PMC13665944; doi:10.1126/sciadv.aei1145)
Supplement: Supplementary file 1 [file sciadv.aei1145_sm.pdf]

Science Advances
AAAS

Supplementary Materials for

Seabirds and native vegetation drive ecosystem processes that support atoll island resilience

Nicholas A. J. Graham *et al.*

Corresponding author: Nicholas A. J. Graham, nick.graham@lancaster.ac.uk

Sci. Adv. **12**, eaei1145 (2026)
DOI: 10.1126/sciadv.aei1145

This PDF file includes:

Figs. S1 to S6
Tables S1 to S3

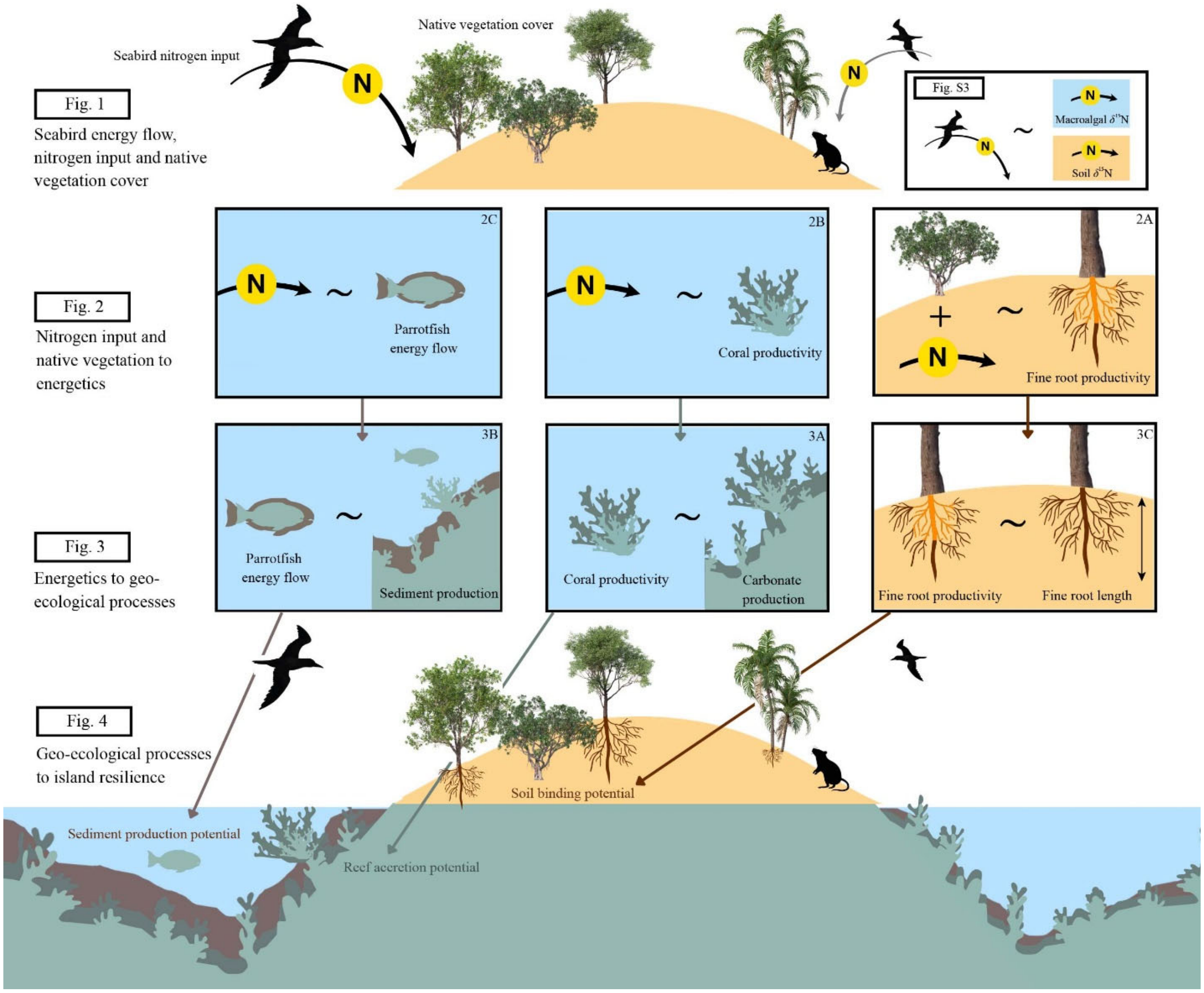

Fig. S1. Heuristic describing study metrics and figure development.

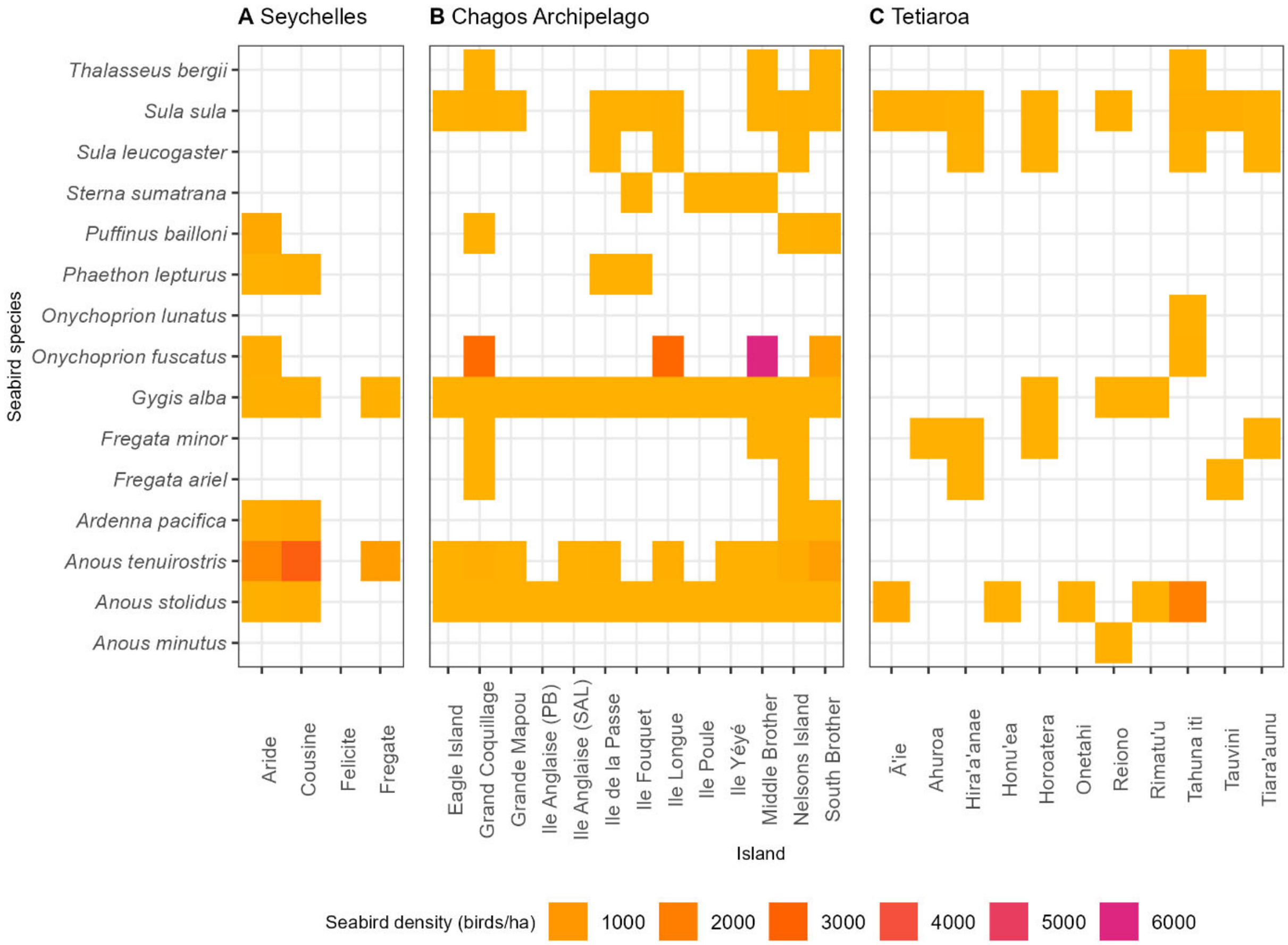

Fig. S2. Seabird composition and density across the 28 study islands.
Densities less than 1 bird per hectare not included in plot, but are included in statistical analyses.

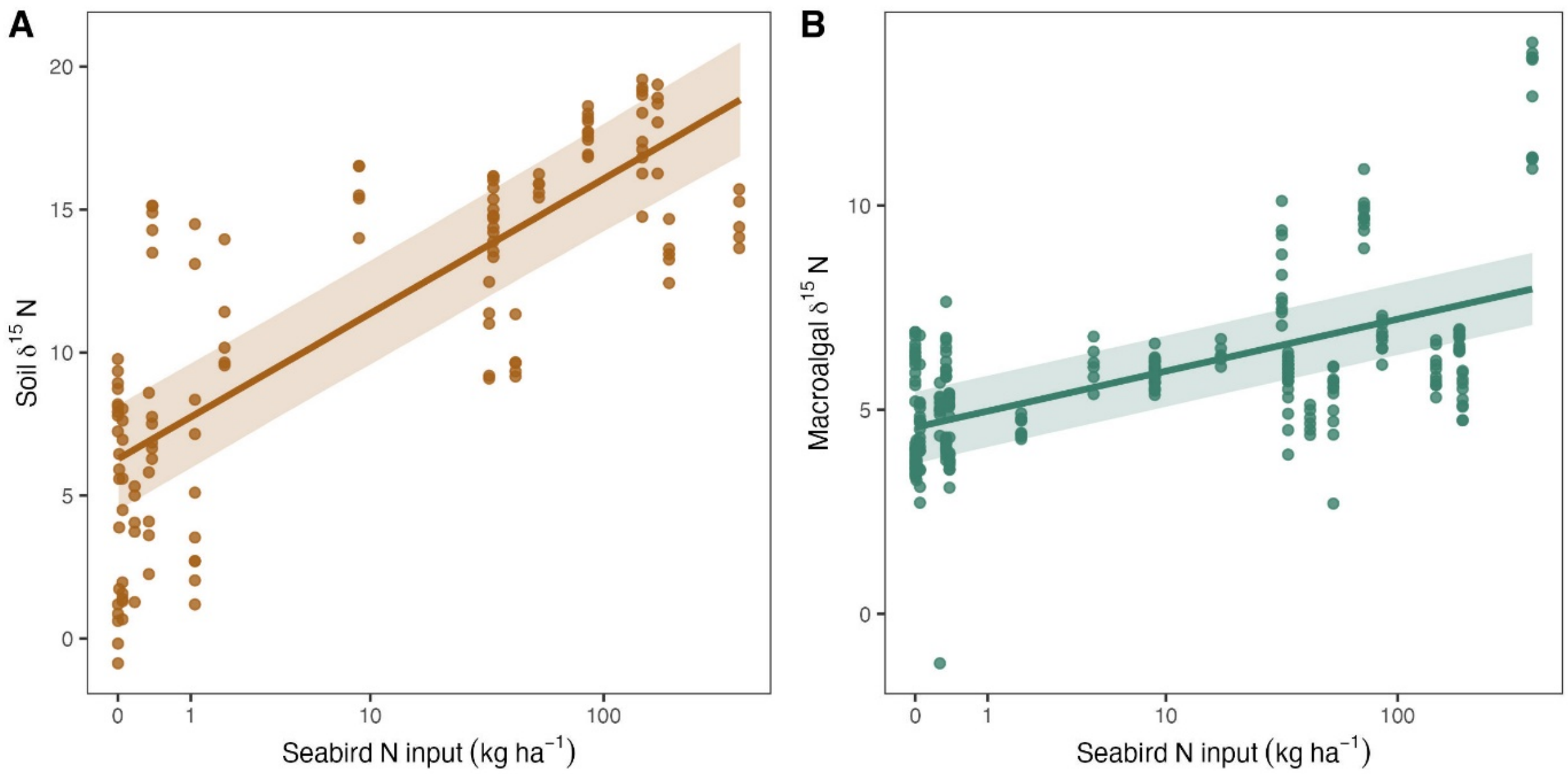

Fig. S3. Relationship between seabird nitrogen input and (A) soil δ^{15}N in forest plots, and (B) macroalgal δ^{15}N in adjacent marine survey sites.

Soil data includes n = 133 cores across 21 islands. Macroalgal data includes 278 samples across 24 islands. All confidence bands around trends are 75% Bayesian credible intervals.

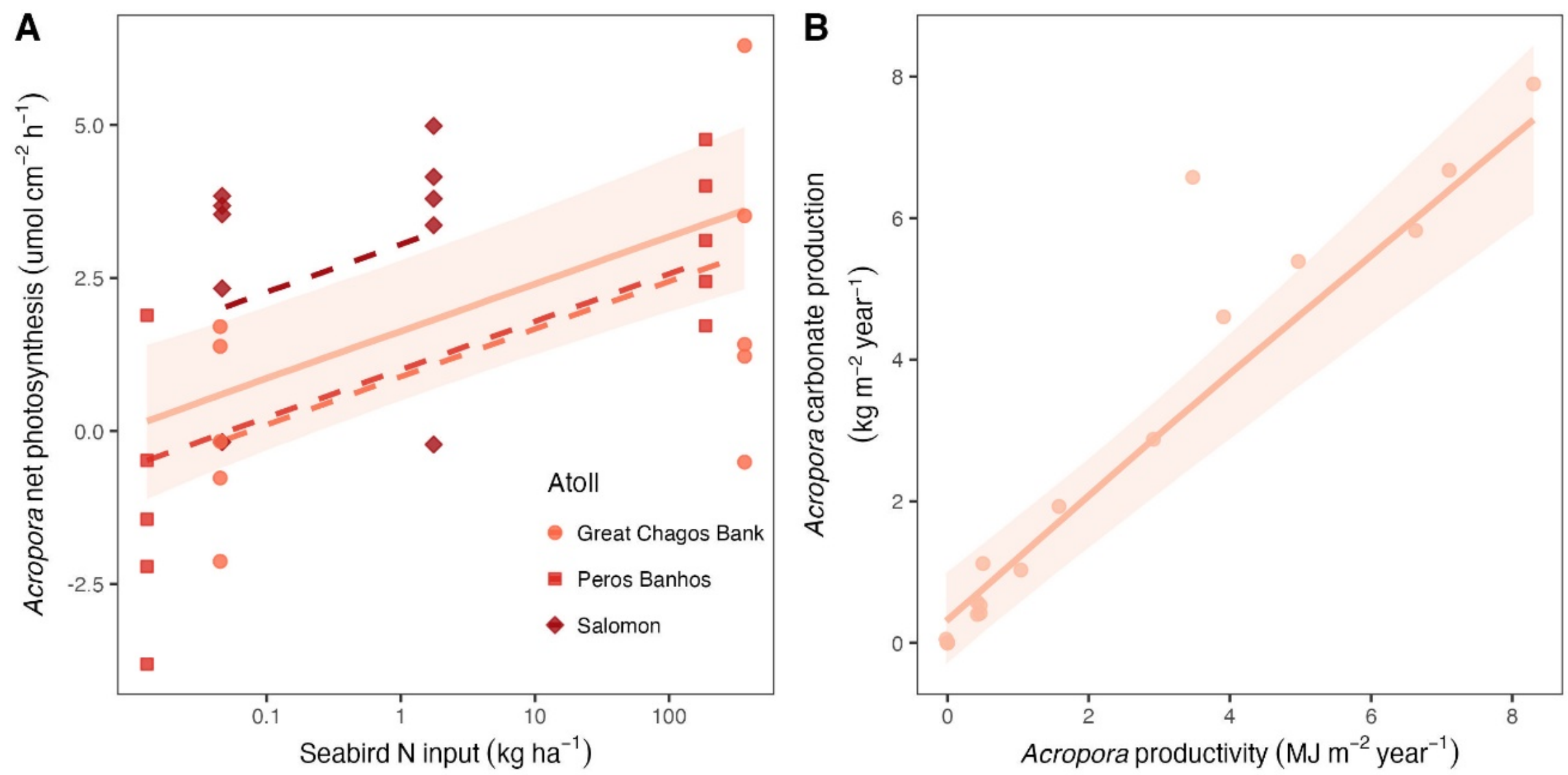

Fig. S4. Scaling of seabird nitrogen input, coral primary productivity and carbonate production of *Acropora* corals. (**A**) Relationship between seabird nitrogen input to islands and coral net photosynthesis on adjacent coral reefs, with 75% Bayesian credible intervals. Solid line = global trend, dotted lines = atoll-level trends due to differing intercepts (n = 30 coral fragments across 6 islands in the Chagos Archipelago). (**B**) *Acropora* net primary productivity calculated from net photosynthesis rates and *Acropora* coral cover against *Acropora* carbonate production calculated from colony-level calcification rates (n = 24 transects across 6 islands in Chagos Archipelago).

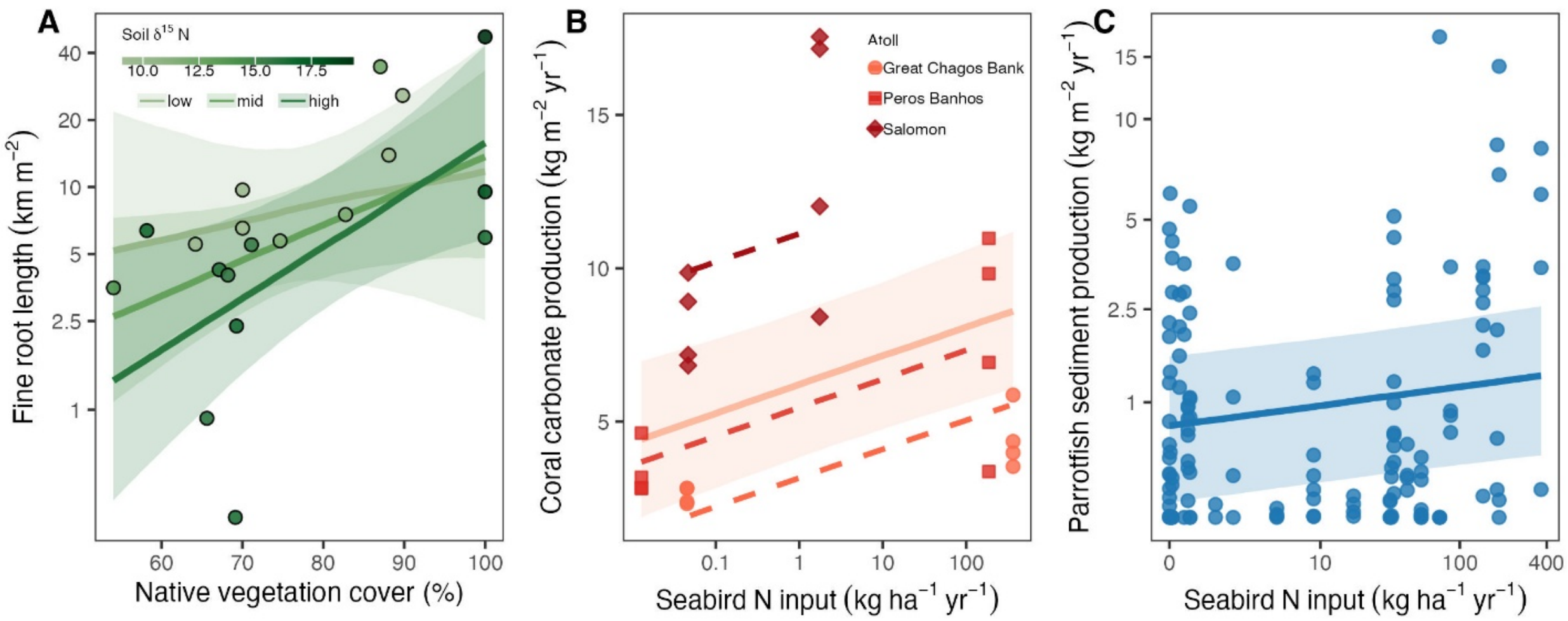

Fig. S5. Key ecological processes and functions on islands and adjacent coral reefs in relation to seabird nutrient inputs and native vegetation cover.
(**A**) Relationship between native vegetation cover and fine root length across three representative soil δ^{15}N values indicative of differing seabird nutrient inputs (low = mean – 1 SD, mid = mean, high = mean + 1 SD) with 75% Bayesian credible intervals (n=20 plots shaded by gradient in soil δ^{15}N across 5 islands in Tetiaroa). (**B**) Relationship between seabird nitrogen input to islands and coral carbonate production on adjacent coral reefs, with 75% Bayesian credible intervals. Solid line = global trend, dotted lines = atoll-level trends due to differing intercepts (n=24 transects across 6 islands in the Chagos Archipelago). (**C**) Relationship between seabird nitrogen input to islands and parrotfish sediment production, with 75% Bayesian credible intervals (n=134 transects across 27 islands in all three study locations).

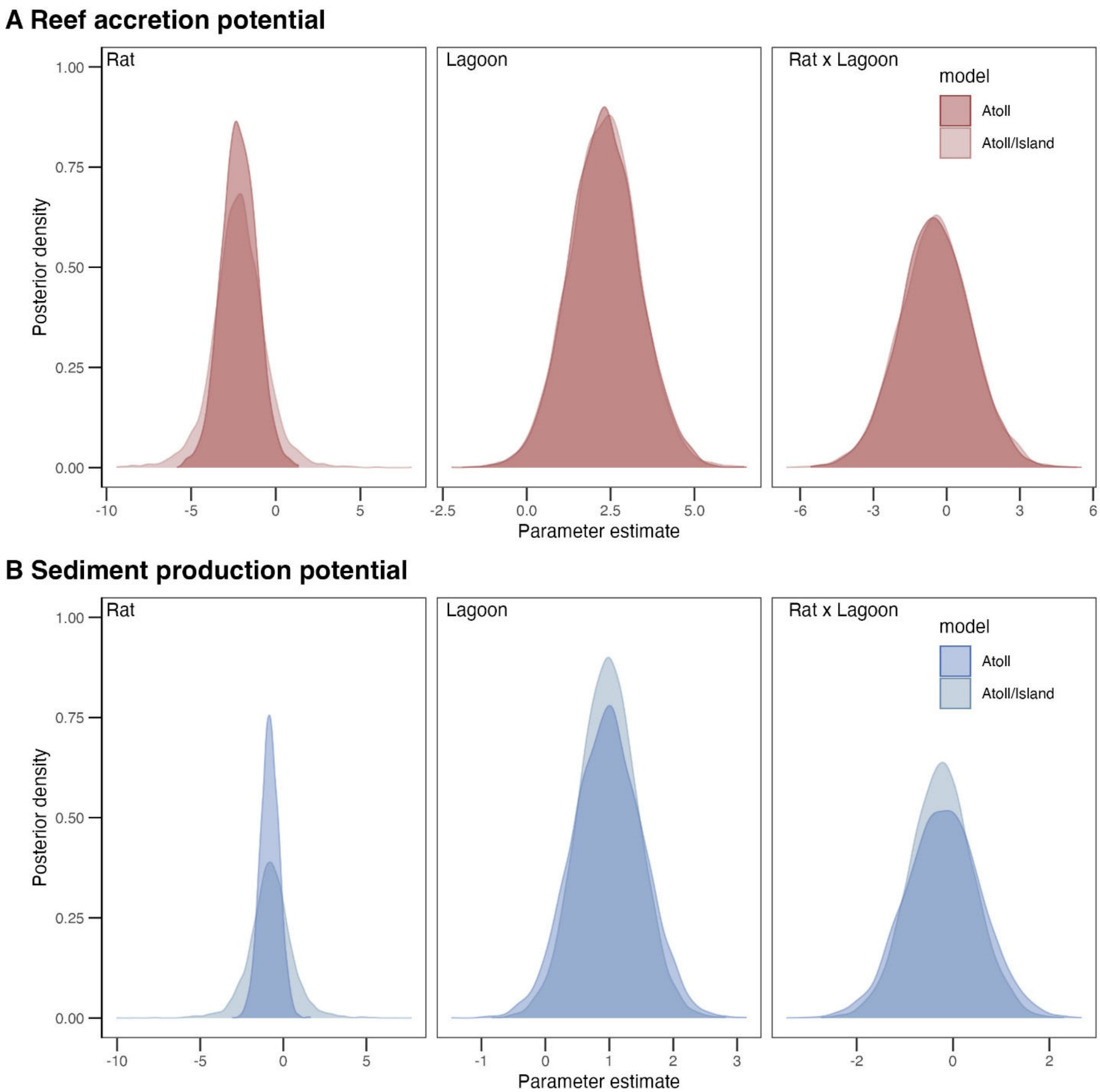

Fig. S6. Sensitivity analysis of reef geo-ecological functions.
Comparison of hierarchical model structure on estimates of (**A**) reef accretion potential and (**B**) sediment production potential on rat-infested verses rat-free, lagoonal versus forereef sites and their interaction in the Chagos Archipelago, comparing models with group level effects at the level of the atoll, and those with island nested in atoll.

Table S1. Distribution of response metric sampling across study geographies. Numbers represent number of islands sampled per geography for each response, with the total replication per geography given in parentheses. Measure column describes data source: S = survey data collection, M = modeled from published parameters, C = chemical analyses, I = inferred from taxa specific rate data.

General response	Specific response	Measure	Seychelles	Chagos Archipelago	Tetiaroa
Seabird energy flow, nitrogen input and vegetation cover	Seabird metrics (Fig 1, Fig S2)	S, M	4(4)	13(13)	11(11)
	Native vegetation (Fig 1)	S	4(4)	13(13)	11(11)
	Soil δ^{15}N (Fig S3A)	S, C	9(45)	4(43)	8(45)
	Macroalgae δ^{15}N (Fig S3B)	S, C	4(64)	12(118)	8(96)
Island and reef energetics	Fine root productivity (Fig 2A)	S, M			5(20)
	Coral productivity (Fig 2B)	S, M		6(24)	
	Parrotfish energy (Fig 2C)	S, M	4(36)	13(52)	10(46)
Geo-ecological processes	Carbonate production (Fig 3A)	S, I		6(24)	
	Sediment production (Fig 3B)	S, I	4(36)	13(52)	10(46)
	Fine root length (Fig 3C)	S			5(19)
Restoration potential	Reef accretion potential (Fig 4A,C)	S, I		6(48)	
	Sediment production potential (Fig 4B,D)	S, I		6(47)	
	Soil binding potential (Fig 4E)	S			5(19)

Table S2. Constituent components of reef accretion potential per site.

Site level means (Kg m^2 yr^{-1} ± SD)	Coral prod-uction	CCA prod-uction	Parrot-fish erosion	Urchin erosion	Macro-boring	Micro-boring	Net prod-uction	Sediment infill	RAPmax (mm yr^{-1})
**Shallow fore-reef sites**									
Ile de la Passe	8.68 ± 3.65	0.33 ± 0.02	1.65 ± 1.12	0.20 ± 0.15	0.08 ± 0.01	0.26 ± 0.03	6.81 ± 3.69	0.55 ± 0.28	5.10 ± 2.53
Grande Coquillage	2.96 ± 0.86	0.21 ± 0.06	0.39 ± 0.61	0.16 ± 0.12	0.09 ± 0.01	0.25 ± 0.01	2.28 ± 0.87	0.22 ± 0.20	1.73 ± 0.56
Middle Brother	8.53 ± 3.19	0.12 ± 0.06	1.14 ± 1.42	0.74 ± 0.82	0.08 ± 0.01	0.28 ± 0.01	6.42 ± 4.60	0.69 ± 0.45	4.92 ± 2.92
Ile Anglaise (SAL)	7.42 ± 2.64	0.60 ± 0.07	4.39 ± 5.57	-	0.08 ± 0.01	0.31 ± 0.03	3.24 ± 4.22	1.14 ± 1.39	3.03 ± 2.10
Ile Poule	2.48 ± 0.22	0.47 ± 0.13	1.69 ± 2.68	0.09 ± 0.11	0.12 ± 0.01	0.32 ± 0.01	0.73 ± 2.77	0.53 ± 0.65	0.87 ± 1.47
Eagle	2.39 ± 0.49	0.04 ± 0.02	0.32 ± 0.65	-	0.09 ± 0.01	0.25 ± 0.03	1.75 ± 0.58	0.13 ± 0.17	1.30 ± 0.34
**Lagoon sites**									
Ile de la Passe	13.79 ± 4.38	0.17 ± 0.03	1.35 ± 0.63	-	0.08 ± 0.02	0.35 ± 0.02	12.17 ± 4.92	0.38 ± 0.17	10.89 ± 4.13
Grande Coquillage	7.78 ± 3.40	0.34 ± 0.05	3.04 ± 1.87	0.08 ± 0.07	0.10 ± 0.01	0.29 ± 0.01	4.60 ± 4.81	0.85 ± 0.49	4.72 ± 3.80
Middle Brother	4.43 ± 1.01	0.05 ± 0.03	0.99 ± 1.48	-	0.07 ± 0.01	0.20 ± 0.01	3.23 ± 2.03	0.28 ± 0.37	3.04 ± 1.48
Ile Anglaise (SAL)	8.39 ± 1.40	0.26 ± 0.10	0.75 ± 0.67	-	0.09 ± 0.02	0.38 ± 0.07	7.24 ± 2.13	0.23 ± 0.16	6.46 ± 1.70
Ile Poule	3.37 ± 0.86	0.46 ± 0.15	1.03 ± 1.33	0.03 ± 0.04	0.10 ± 0.01	0.29 ± 0.02	2.37 ± 1.88	0.32 ± 0.35	2.33 ± 1.37
Eagle	2.59 ± 0.27	0.24 ± 0.06	0.43 ± 0.69	-	0.09 ± 0.01	0.24 ± 0.01	2.08 ± 0.93	0.15 ± 0.17	1.93 ± 0.66

Table S3. Soil binding potential depicted by fine root length as a function of native forest and rat-eradication targets.

Native forest cover	Rat status	Mean fine root length (km m^{-2})	Median fine root length (km m^{-2})	n	Standard deviation
Over 75%	No rats	20.9	9.5	3	22.9
Over 75%	Rats	20.5	19.8	4	12.1
Under 75%	No rats	3.4	3.8	8	2.1
Under 75%	Rats	6.9	6.1	4	1.9
